# Prognostic Role of Left Atrial Reservoir Strain for Risk Stratification in Aortic Stenosis: A Systematic Review

**DOI:** 10.3390/jcm15114304

**Published:** 2026-06-02

**Authors:** Andrea Sonaglioni, Massimo Baravelli, Giulio Francesco Gramaglia, Gian Luigi Nicolosi, Michele Lombardo

**Affiliations:** 1 Division of Cardiology, IRCCS MultiMedica, 20123 Milan, Italy; massimo.baravelli@multimedica.it (M.B.); michele.lombardo@multimedica.it (M.L.); 2 Department of Emergency, Fondazione IRCSS Ca’ Granda, Ospedale Maggiore Policlinico, 20122 Milan, Italy; giulio.gramaglia@unimi.it; 3 Division of Cardiology, Policlinico San Giorgio, 33170 Pordenone, Italy; gianluigi.nicolosi@gmail.com

**Keywords:** aortic stenosis, left atrial reservoir strain, prognosis, speckle tracking echocardiography, cardiac computed tomography, cardiac magnetic resonance

## Abstract

**Background:** Risk stratification in aortic stenosis (AS) remains challenging, particularly in patients with preserved left ventricular ejection fraction or inconclusive symptom status, as conventional parameters primarily reflect valvular obstruction and may underestimate the extent of cardiac dysfunction. Left atrial reservoir strain (LASr) has emerged as a promising and potentially more comprehensive marker of atrial function and diastolic burden, with potential prognostic implications. **Methods:** A systematic review was conducted in accordance with PRISMA guidelines. PubMed, Scopus, and EMBASE were searched from inception to April 2026. Studies including adult patients with moderate or severe AS and evaluating LASr using different imaging modalities (speckle-tracking echocardiography, cardiac computed tomography, or cardiac magnetic resonance) were considered eligible if clinical outcomes were reported. Data were qualitatively synthesized, and continuous variables were summarized as weighted medians with interquartile ranges. **Results:** Twenty-one studies were included, encompassing a large and clinically heterogeneous population. During follow-up, a substantial proportion of patients experienced adverse events, including mortality, heart failure hospitalization, arrhythmic events, and composite cardiovascular outcomes. Across studies, reduced LASr consistently emerged as a significant predictor of adverse outcomes. This association was observed both when LASr was analyzed as a continuous variable and when defined using study-specific cut-off values, which generally clustered within a relatively narrow range. Importantly, LASr demonstrated incremental prognostic value beyond conventional echocardiographic parameters, including left atrial size, left ventricular ejection fraction, global longitudinal strain, and indices of diastolic dysfunction. The prognostic relevance of LASr was consistent across different imaging modalities, including both echocardiography and cardiac computed tomography (with no eligible studies using cardiac magnetic resonance). **Conclusions:** LASr is a robust and reproducible marker of adverse prognosis in patients with AS, reflecting the cumulative burden of left-sided pressure overload and atrial remodeling. Its integration into multiparametric assessment may enhance risk stratification and support more individualized clinical decision-making. Further prospective studies are warranted to standardize measurement techniques and define clinically actionable thresholds.

## 1. Introduction

Aortic stenosis (AS) is the most common valvular heart disease in developed countries [1] and represents a major cause of cardiovascular morbidity and mortality, particularly in the aging population [2]. Its clinical course is characterized by a prolonged asymptomatic phase followed by rapid clinical deterioration once symptoms develop, making the identification of early markers of disease progression a central challenge in contemporary cardiology.

Current management strategies rely largely on symptom onset and conventional echocardiographic parameters, such as aortic valve area (AVA) and transvalvular gradients, to guide the timing of intervention [3,4]. However, these indices primarily reflect valvular obstruction and do not fully capture the complex myocardial and hemodynamic consequences of the disease. As a result, patients may remain clinically stable despite ongoing subclinical cardiac remodeling, with potential implications for long-term outcomes [5].

Chronic pressure overload in AS induces a cascade of structural and functional changes involving not only the left ventricle but also the left atrium and the pulmonary circulation [6,7]. While left ventricular adaptation has been extensively studied, increasing attention has been directed toward the left atrium as an early and sensitive marker of cardiac dysfunction [8]. In this context, left atrial remodeling reflects the cumulative effects of elevated filling pressures, impaired diastolic function, and reduced ventricular compliance [9].

Left atrial reservoir strain (LASr), assessed through speckle-tracking echocardiography (STE) and, more recently, cardiac computed tomography (CCT), and potentially by cardiac magnetic resonance (CMR) using feature-tracking (FT) techniques, has emerged as a reproducible and quantitative measure of atrial function [10]. Unlike conventional volumetric parameters, LASr provides a direct assessment of atrial compliance and reservoir capacity, offering incremental insight into the hemodynamic burden imposed by AS [11]. Importantly, impairment of LASr may precede overt changes in left ventricular (LV) systolic function, making it a potential early marker of disease progression [12,13].

Over the past decade, several studies have explored the prognostic significance of LASr in patients with AS across different clinical scenarios, including asymptomatic individuals, patients undergoing transcatheter or surgical valve intervention, and those with varying degrees of ventricular function. These investigations have consistently suggested that reduced LASr is associated with an increased risk of adverse outcomes, including mortality, heart failure hospitalization, and arrhythmic events, as also summarized in recent review articles synthesizing the available prognostic evidence [14,15].

In addition to LASr, other imaging-derived parameters—such as left ventricular global longitudinal strain (LV-GLS) [16], indices of diastolic dysfunction [17], and markers of atrial enlargement [18]—have been proposed as complementary prognostic indicators. Clinical variables, including age [19], comorbidities [20], and biomarkers such as natriuretic peptides [21], further contribute to risk stratification, underscoring the multifactorial nature of disease progression in AS.

Nevertheless, the available evidence remains heterogeneous, with considerable variability in study design, imaging methodologies, LASr cut-off values, and endpoint definitions. This heterogeneity not only limits the direct comparability of individual studies, but also represents a major barrier to the clinical translation of LASr into standardized risk stratification algorithms.

In light of these considerations, a comprehensive synthesis of the current evidence is warranted. The present systematic review was designed to address this critical knowledge gap by providing a structured and up-to-date evaluation of the prognostic role of LASr in patients with AS. By integrating findings across different imaging modalities and clinical settings, this study aims to clarify the consistency and strength of the association between LASr and adverse outcomes, as well as to explore its potential incremental value within a multiparametric risk stratification framework.

Furthermore, this work seeks to characterize the clinical and echocardiographic profiles of the studied populations and to identify the most relevant determinants of prognosis, with the ultimate goal of better defining the role of LASr as a clinically actionable biomarker in contemporary management of AS.

## 2. Materials and Methods

This systematic review was conducted in accordance with the Preferred Reporting Items for Systematic Reviews and Meta-Analyses (PRISMA) guidelines [22] (Supplementary Materials File S1). The study protocol was prospectively registered in April 2026 on the International Platform of Registered Systematic Review and Meta-analysis Protocols (INPLASY) [1,2,3,4,5,6,7,8,9,10,11,12,13,14,15,16,17,18,19,20,21] (registration number: INPLASY202640099; registration date: 27 April 2026; Supplementary Materials File S2).

The overall study design, eligibility criteria, and analytical methods were defined a priori and consistently implemented throughout all phases of the review process.

### 2.1. Literature Search Strategy

A systematic and comprehensive literature search was independently carried out by two investigators to identify studies assessing the prognostic significance of LASr in patients with AS. Studies including adult populations across the full spectrum of disease severity were considered eligible, in order to reflect real-world clinical heterogeneity.

Electronic databases, namely PubMed, Scopus, and EMBASE, were searched from their inception through April 2026. The search strategy integrated both controlled vocabulary and free-text terms related to AS and atrial deformation imaging, including “aortic stenosis”, “left atrial strain”, “left atrial reservoir strain”, “speckle-tracking echocardiography”, “cardiac computed tomography”, “cardiac magnetic resonance”, “feature tracking”, “cardiac deformation”, “prognosis”, “clinical outcome”, and “risk prediction”. Studies employing different imaging modalities for LASr assessment, including echocardiography, CCT, and CMR, were considered.

No restrictions were applied with regard to language, publication year, or geographic location. Furthermore, to ensure completeness of the evidence base, the reference lists of all selected articles and relevant review papers were manually examined to identify additional studies.

Any disagreements between reviewers during the screening and selection process were resolved by discussion and consensus, with adjudication by a third reviewer when necessary.

### 2.2. Study Eligibility Criteria

Studies were considered eligible if they fulfilled predefined inclusion criteria. In particular, only observational studies, including both prospective and retrospective cohort designs, were included. Eligible studies were required to enroll adult patients with moderate or severe AS and to assess LASr using validated imaging techniques, such as two-dimensional (2D) STE, CCT-FT or CMR-FT.

Furthermore, studies had to report clinical outcomes or prognostic endpoints, including all-cause mortality, cardiovascular mortality, heart failure hospitalization, arrhythmic events, or composite cardiovascular outcomes. Only studies providing extractable data on the association between LASr and clinical outcomes were considered.

To ensure methodological consistency, studies focusing exclusively on diagnostic performance without reporting prognostic data were excluded. Additional exclusion criteria included studies with mixed valvular populations without separate analysis of AS, lack of clearly defined cohorts, or absence of relevant outcome data.

Case reports, editorials, conference abstracts, narrative reviews, and experimental or preclinical studies were not eligible for inclusion.

### 2.3. Study Screening and Data Extraction

Study selection was performed independently by two reviewers. All retrieved records were initially screened based on title and abstract, followed by full-text evaluation of potentially eligible studies according to the predefined inclusion criteria. Disagreements were resolved by consensus, with arbitration by a third reviewer when necessary.

Data extraction was carried out using a standardized form developed prior to study initiation. Extracted information included study characteristics such as first author, year of publication, country, study design, imaging modality, and analysis software, as well as sample size and demographic features of the study population.

Clinical and baseline variables were systematically collected when available, including age, sex distribution, anthropometric parameters, cardiovascular risk factors, comorbidities, and ongoing medical therapy.

Echocardiographic and imaging data were extracted in detail, focusing on parameters of LV structure and function, including ventricular dimensions, volumes, mass, and systolic performance assessed by left ventricular ejection fraction (LVEF) and LV-GLS. Diastolic function and filling pressures were evaluated using Doppler-derived indices, particularly the E/e’ ratio. In addition, markers of AS severity, such as AVA and transvalvular pressure gradients, were systematically recorded.

Left atrial structure and function were comprehensively assessed. Structural parameters included left atrial size and volumetric indices, particularly left atrial diameter and left atrial volume index, when available. Functional assessment was performed through myocardial deformation parameters, including left atrial reservoir, conduit, and contractile strain, as well as strain rate indices. Additional deformation measures, such as LV strain and right atrial strain parameters, were also collected when reported.

Outcome data were extracted for all included studies, including duration of follow-up, definition of clinical endpoints, and the main prognostic predictors identified in each analysis. Particular attention was given to the association between LASr and adverse outcomes, including both continuous and categorical (cut-off-based) analyses.

All extracted data were independently verified by both reviewers, and any discrepancies were resolved through re-evaluation of the original articles.

### 2.4. Assessment of Methodological Quality and Risk of Bias

The methodological quality and risk of bias of the included studies were independently assessed by two reviewers using the National Institutes of Health (NIH) Quality Assessment Tool for Observational Cohort and Cross-Sectional Studies [23].

This instrument evaluates multiple domains of internal validity, including the clarity of the research question, definition of the study population, adequacy of exposure and outcome assessment, consistency of measurement methods, control of confounding factors, and appropriateness of statistical analyses. Each item was rated as “Yes”, “No”, or “Not Reported/Not Applicable”, according to predefined criteria.

An overall quality rating (Good, Fair, or Poor) was assigned to each study based on the number of criteria fulfilled and the overall methodological rigor. Discrepancies between reviewers were resolved through discussion and consensus.

The results of the quality assessment were summarized both descriptively and through graphical representations, including a traffic-light plot and a risk-of-bias summary, to provide a comprehensive overview of methodological quality across studies.

### 2.5. Evidence Synthesis and Analytical Framework

The substantial variability across studies in terms of design, imaging techniques (including both echocardiography and computed tomography), patient characteristics, and outcome definitions precluded the performance of a formal quantitative meta-analysis. In particular, considerable heterogeneity was observed regarding the definition of clinical endpoints, duration of follow-up, LASr acquisition techniques, imaging software, statistical modeling strategies, and reporting of prognostic estimates. Moreover, studies variably analyzed LASr as a continuous parameter or according to different study-specific cut-off values, frequently using non-uniform adjustment models and inconsistent reporting of hazard ratios, confidence intervals, or standard errors. These methodological differences limited the feasibility and reliability of pooled quantitative analyses, even within potential study subgroups. Therefore, the available evidence was integrated through a structured qualitative and descriptive approach.

To summarize baseline characteristics, aggregate estimates were derived from study-level data. Continuous variables were expressed as weighted medians with corresponding interquartile ranges (IQR), applying weights proportional to the sample size of each study. When data were reported as mean and standard deviation, approximate distributions were inferred to ensure comparability across datasets. Specifically, in studies reporting continuous variables as mean ± standard deviation, approximate median and dispersion measures were derived under the assumption of near-normal data distribution, allowing harmonization with studies reporting medians and IQRs. Weighting procedures were based exclusively on the total sample size of each study to provide proportionally greater influence to larger cohorts. This descriptive strategy was selected to reduce the potential distortion that could arise from extreme values, heterogeneous reporting methods, and substantial inter-study variability, while still providing an overall representation of the included populations. Variables with insufficient, incomplete, or non-comparable reporting across studies were not aggregated and were instead summarized descriptively. No formal imputation techniques were applied for missing data. Given the descriptive and non-meta-analytic nature of this synthesis, no formal sensitivity analyses were performed; however, the consistency of pooled descriptive estimates was qualitatively verified across studies with different sample sizes and clinical settings. This approach should be regarded as an approximate descriptive strategy and may be influenced by assumptions regarding data distribution and inter-study heterogeneity. Accordingly, pooled descriptive estimates should be interpreted as exploratory summaries rather than definitive quantitative effect measures.

This methodology enabled a harmonized synthesis of demographic, clinical, and imaging-derived variables, including measures of AS severity, LV function, and left atrial mechanics. Particular attention was devoted to LASr, alongside additional phasic strain components and indices of myocardial deformation.

Considering the observational design of the included studies, clinical endpoints and prognostic determinants were analyzed descriptively rather than quantitatively. Identified predictors were interpreted within their underlying pathophysiological context and categorized into key domains, including left atrial function, LV performance, valvular severity, clinical characteristics, and circulating biomarkers. The frequency with which these variables were reported across studies was also evaluated to highlight the most consistently observed prognostic markers.

Where available, effect size estimates (hazard ratios or odds ratios) and study-specific LASr threshold values were reported descriptively to provide an overall perspective on the prognostic relevance of impaired left atrial function.

No formal meta-analytic pooling, heterogeneity testing, or assessment of publication bias was undertaken. The decision to avoid formal quantitative pooling was also supported by the limited methodological comparability across studies and by the absence of sufficiently homogeneous datasets suitable for reliable statistical aggregation. Nonetheless, the consistency, direction, and reproducibility of associations across studies were carefully examined to support the validity of the overall interpretation.

All analyses were conducted at the study level. Data handling and aggregation were performed using standard spreadsheet software (Microsoft Excel, Microsoft Corporation, Redmond, WA, USA).

### 2.6. Artificial Intelligence-Supported Language Editing

Artificial intelligence-based tools were utilized solely to assist in linguistic editing during the preparation of the manuscript. In particular, ChatGPT (OpenAI, San Francisco, CA, USA; version GPT-5.3) was used to enhance grammatical accuracy, improve clarity, and optimize overall readability of the text. Its use was strictly limited to language refinement and editorial support.

No artificial intelligence applications were involved in the conception of the study, data extraction, statistical analyses, or interpretation of the findings. All scientific content, methodological decisions, and conclusions were independently conceived, critically reviewed, and validated by the authors.

## 3. Results

### 3.1. Study Identification and Selection Process

The literature search identified 359 records from PubMed, Scopus, and EMBASE databases. After removal of duplicates (n = 26), 333 articles remained for screening. Following title and abstract evaluation, 290 records were excluded based on predefined criteria. The remaining 43 studies underwent full-text assessment for eligibility. Of these, 22 articles were excluded due to insufficient prognostic data. Ultimately, 21 studies [24,25,26,27,28,29,30,31,32,33,34,35,36,37,38,39,40,41,42,43,44] met the inclusion criteria and were incorporated into the final analysis.

**Figure 1:**
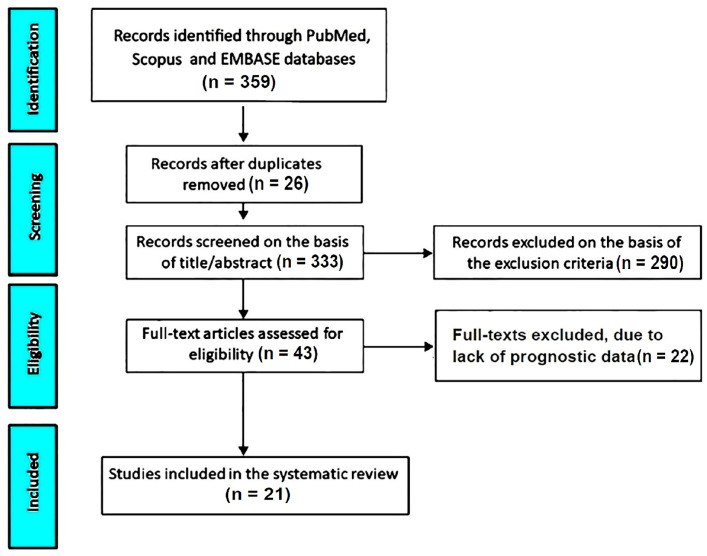
Overview of the literature search and study selection process.

The study selection process is summarized in Figure 1.

### 3.2. Overview of Included Studies

The main characteristics of the studies included in this systematic review are summarized in Table 1.

Overall, 21 studies published between 2014 and 2026 were analyzed, encompassing both prospective and retrospective observational designs.

Most investigations were conducted in single-center settings, with only a limited number of multicenter studies. Despite some heterogeneity in study design, the overall methodological framework was relatively consistent, particularly regarding imaging acquisition and strain analysis.

Left atrial reservoir strain was predominantly assessed using 2D-STE, representing the primary imaging modality across the majority of studies. A smaller subset of investigations employed CCT with FT techniques, supporting the feasibility of multimodality strain assessment. Notably, no studies evaluating the prognostic role of LASr using CMR-FT met the inclusion criteria. Various vendor-specific software platforms were used, including GE, TomTec, Philips, Siemens, and Medis, reflecting real-world variability in strain analysis.

The study populations were generally composed of patients with moderate or severe AS, with mean ages typically in the seventh to eighth decade of life. Across studies, the proportion of male participants was consistently high, generally around or above 50%, indicating a slight predominance of male subjects.

Sample sizes varied substantially, ranging from small cohorts of fewer than 100 patients to large observational studies including more than 1000 individuals. Aortic valve area values were reported in most studies and reflected a spectrum of disease severity, from moderate to severe AS.

Prognostic thresholds for LASr were reported in the majority of studies and showed a relatively narrow distribution, typically ranging between approximately 15% and 22%, although some variability was observed depending on population characteristics and outcome definitions.

Taken together, the included studies provide a comprehensive representation of different clinical scenarios in AS, encompassing a broad range of disease severity, imaging approaches, and patient profiles, while maintaining sufficient methodological consistency to support comparative evaluation of LASr as a prognostic marker.

### 3.3. Baseline Characteristics of the Study Population

The pooled baseline characteristics of the study population are summarized in Table 2.

In total, the analysis included a large cohort of patients derived from the selected studies, representing a predominantly elderly population with a slight male predominance.

Anthropometric data, when available, suggested relatively homogeneous body size characteristics, overall consistent with a mildly overweight profile. However, these parameters were not uniformly reported across all studies.

Hemodynamic variables were inconsistently described, but when available, they generally fell within normal to mildly elevated ranges, reflecting a relatively stable baseline clinical condition.

Cardiovascular risk factors were common across the included cohorts. Hypertension emerged as the most frequently reported comorbidity, followed by dyslipidemia and diabetes mellitus. A history of smoking was also frequently documented. Coronary artery disease was present in a substantial proportion of patients, whereas prior cerebrovascular events were less commonly reported.

Clinical presentation varied among studies, with a notable proportion of patients exhibiting advanced symptoms. Atrial fibrillation and heart failure were also recurrent findings, although their prevalence differed across cohorts.

Additional comorbidities, including chronic kidney disease and chronic obstructive pulmonary disease, were reported in several studies, albeit with variable consistency. Renal function, when described, indicated a degree of impairment in a subset of patients.

Biomarker data were available only in selected cohorts. Natriuretic peptide levels showed considerable variability, reflecting heterogeneous degrees of cardiac stress within the population.

Pharmacological therapy was generally in line with contemporary cardiovascular management. Renin–angiotensin system inhibitors and beta-blockers were widely used, along with diuretics and lipid-lowering agents in a substantial proportion of patients. Other treatments were less consistently reported, likely reflecting differences in clinical status and study design.

Collectively, the study population reflects a clinically heterogeneous spectrum of patients with AS, characterized by advanced age, a moderate burden of cardiovascular comorbidities, and variable clinical presentation. This heterogeneity provides a solid basis for evaluating the prognostic relevance of LASr across diverse clinical settings.

### 3.4. Baseline Transthoracic Echocardiographic Findings

Table 3 illustrates the main transthoracic echocardiographic parameters of the study population.

At baseline, LV systolic function was generally preserved or only mildly impaired, indicating an overall compensated functional status.

Left ventricular geometry and remodeling indices were consistent with chronic pressure overload, including increased wall thickness and relative wall thickness, together with mildly enlarged ventricular volumes. These findings are indicative of adaptive structural changes related to AS.

Diastolic function parameters showed varying degrees of impairment across studies. In particular, indices of LV filling pressure were frequently elevated, suggesting impaired relaxation and increased stiffness. This was accompanied by evidence of left atrial remodeling, with enlarged atrial volumes commonly reported.

Hemodynamic indices of AS severity confirmed the presence of moderate-to-severe disease, as reflected by elevated transvalvular gradients and reduced AVA.

Right ventricular function was largely preserved at rest, based on longitudinal functional indices. However, pulmonary artery pressures were often mildly increased, indicating early involvement of the pulmonary circulation secondary to elevated left-sided pressures.

A proportion of patients also presented with concomitant valvular abnormalities, including mild degrees of aortic, mitral, and tricuspid regurgitation, although these were generally not hemodynamically significant.

Overall, the echocardiographic profile reflects a population characterized by preserved systolic function, structural remodeling of the left ventricle and atrium, and varying degrees of diastolic dysfunction in the setting of AS.

### 3.5. Left Atrial Strain and Myocardial Deformation Parameters

The distribution of left atrial strain and myocardial deformation indices across the included studies is detailed in Table 4.

Among atrial functional parameters, LASr was the most consistently reported measure, available in the vast majority of studies, followed by conduit and contractile strain.

Overall, atrial strain values reflected a variable degree of left atrial dysfunction across the study population. Reservoir function appeared moderately impaired, while conduit and contractile components showed a broader dispersion, suggesting heterogeneity in atrial phasic function depending on disease severity and clinical setting.

When stratified by imaging modality, weighted mean LASr values were lower in studies using CCT with FT compared with STE (approximately 16.7% vs. 20.9%). This systematic difference is consistent with previously reported methodological variability and likely reflects differences in temporal resolution, image acquisition, and post-processing algorithms between CCT and echocardiographic techniques, rather than true physiological discrepancies.

**Figure 2:**
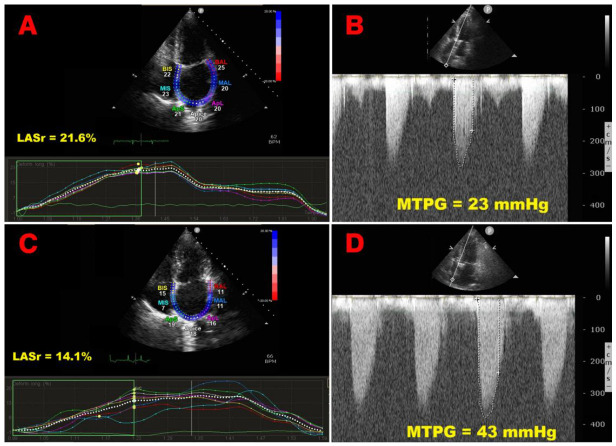
Representative Assessment of Left Atrial Reservoir Strain in Moderate and Severe Aortic Stenosis. Panels (**A**,**B**) show a case of moderate aortic stenosis, characterized by relatively preserved LASr values (21.6%) and a lower mean transvalvular pressure gradient (MTPG = 23 mmHg). Panels (**C**,**D**) depict a case of severe aortic stenosis, with markedly reduced LASr (14.1%) and a higher transvalvular gradient (MTPG = 43 mmHg). Panels (**A**,**C**) display speckle-tracking analysis of the left atrium from the apical view, including segmental strain curves and global LASr values. Panels (**B**,**D**) show continuous-wave Doppler recordings across the aortic valve used to derive the mean transvalvular pressure gradient. These examples illustrate the progressive impairment of left atrial reservoir function with increasing severity of aortic stenosis. LASr, left atrial reservoir strain; MTPG, mean transaortic pressure gradient.

Figure 2 provides a representative illustration of LASr assessment in two patients with different degrees of AS severity.

Strain rate parameters were less frequently reported but provided complementary insights into atrial mechanics. When available, systolic and diastolic strain rate indices supported the presence of impaired atrial compliance and altered reservoir function.

In addition to atrial deformation, LV strain parameters were reported in several studies. Global longitudinal strain was generally reduced compared to normal reference values [45], indicating subclinical impairment of myocardial function despite preserved ejection fraction in a large proportion of patients.

Limited data were available for other deformation indices, including circumferential and radial strain, as well as right atrial strain parameters. These measures were reported only in a small subset of studies, precluding comprehensive comparisons but suggesting a potential extension of myocardial involvement beyond the left atrium.

Composite indices integrating atrial function and filling pressures, such as the ratio between E/e’ and LASr, were only sporadically evaluated, although they may provide additional pathophysiological insights into atrioventricular coupling.

In summary, myocardial deformation analysis highlights the presence of both atrial and ventricular functional impairment, with left atrial strain emerging as a widely available and reproducible marker of cardiac remodeling in patients with AS.

### 3.6. Clinical Outcomes and Determinants of Prognosis

Table 5 reports follow-up duration, event rates, and the main prognostic determinants identified across the included studies.

Follow-up length varied considerably among studies, ranging from short-term observational periods to extended longitudinal follow-up, reflecting differences in study design and clinical settings. Event rates were similarly heterogeneous, with a substantial proportion of patients experiencing adverse outcomes during follow-up.

The most frequently reported endpoints included all-cause and cardiovascular mortality, heart failure hospitalization, and composite outcomes incorporating valve intervention and major adverse cardiovascular events. Additional outcomes, such as new-onset atrial fibrillation and post-procedural complications, were also evaluated in selected cohorts.

Across studies, LASr consistently emerged as a key prognostic marker. Reduced LASr values were associated with an increased risk of adverse outcomes, whether analyzed as a continuous variable or using predefined cut-off thresholds. Notably, LASr demonstrated independent prognostic value in multiple studies, often outperforming or complementing conventional echocardiographic and clinical parameters.

Cut-off values for LASr showed a relatively narrow distribution across studies, typically clustering around clinically relevant thresholds, supporting its potential role in risk stratification. In addition, other components of atrial function, including conduit strain and strain rate parameters, were identified as predictors in selected analyses, although less consistently than LASr.

Beyond atrial deformation, several additional variables contributed to prognostic stratification. Measures of LV function, particularly GLS, were frequently associated with outcomes, reflecting the presence of subclinical myocardial dysfunction. Diastolic indices, including elevated filling pressures, and markers of left atrial enlargement also demonstrated prognostic relevance in selected studies.

Clinical variables further refined risk assessment. Age, comorbidity burden, atrial fibrillation, diabetes mellitus, and biomarkers such as natriuretic peptides were recurrently identified as significant predictors. In some studies, composite indices integrating clinical and imaging variables provided additional prognostic information.

**Figure 3:**
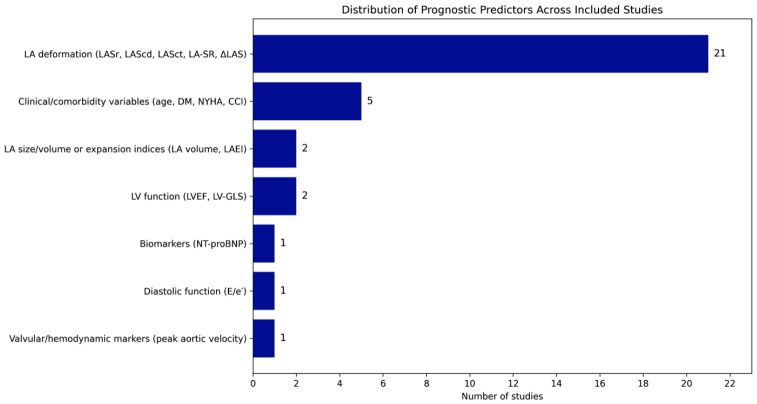
Bar chart illustrating the frequency with which different categories of prognostic variables were reported across the included studies. Left atrial deformation parameters represent the most consistently investigated domain, followed by clinical and comorbidity-related variables. Structural indices, ventricular function parameters, biomarkers, and hemodynamic markers were less frequently evaluated. CCI, Charlson Comorbidity Index; DM, diabetes mellitus; LA, left atrial; LAEI, left atrial expansion index; LAScd, left atrial conduit strain; LASct, left atrial contractile strain; LASr, left atrial reservoir strain; LV, left ventricular; LV-GLS, left ventricular global longitudinal strain; LVEF, left ventricular ejection fraction; NYHA, New York Heart Association.

The distribution of prognostic markers across included studies is illustrated in Figure 3, which highlights the predominance of left atrial deformation parameters as the most consistently evaluated domain. Other categories, including clinical variables, ventricular function, and structural indices, were less frequently represented, underscoring the central role of atrial mechanics in the prognostic assessment of AS.

**Figure 4:**
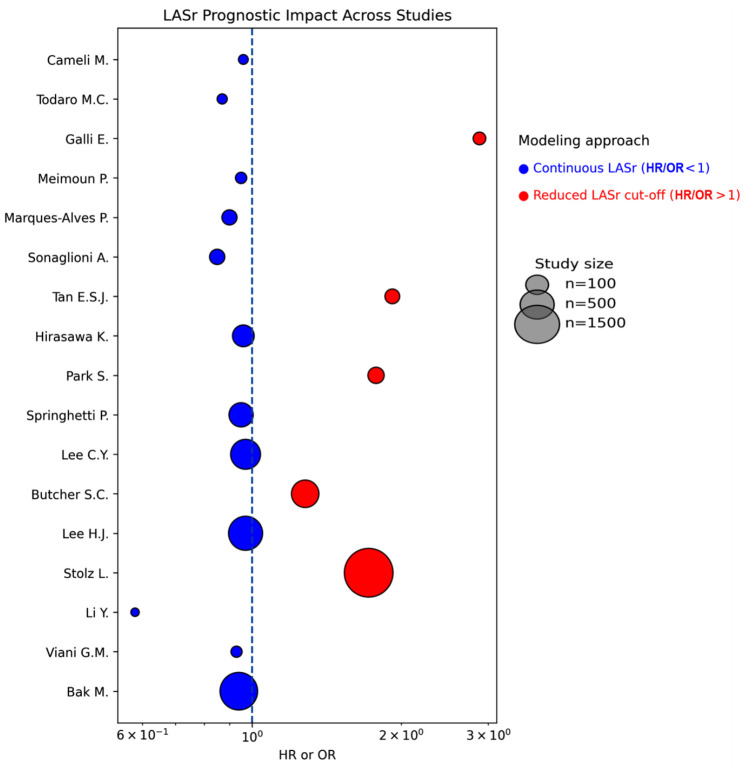
Prognostic Impact of Left Atrial Reservoir Strain Across Studies [24,26,27,29,30,31,33,34,35,37,38,39,40,41,42,43,44]. Bubble plot displaying effect estimates of LASr across individual studies. Two analytical approaches are shown: continuous models (blue), in which LASr was analyzed as a continuous variable, and categorical models based on predefined LASr cut-off values (red). In general, lower LASr values or reduced LASr categories were associated with a higher risk of adverse clinical outcomes. The vertical dashed line represents the null value (HR/OR = 1). Bubble size is proportional to study sample size. Given the heterogeneity in statistical methodology, endpoint definitions, and multivariable adjustment strategies across studies, the figure should be interpreted as a qualitative representation of the overall direction and consistency of prognostic associations rather than as a direct quantitative comparison of effect estimates. HR, hazard ratio; LASr, left atrial reservoir strain; OR, odds ratio.

Figure 4 depicts the distribution of effect estimates for LASr across individual studies, taking into account the different modeling approaches used.

In several studies, LASr was modeled as a continuous variable, showing that lower LASr values are associated with a higher risk of adverse events (HR < 1), thus reflecting a protective effect of preserved atrial reservoir function. In contrast, when LASr is analyzed using predefined cut-off values (e.g., LASr < 20%) as a categorical variable, higher risk is observed in patients with reduced LASr (HR > 1), indicating worse outcomes below these thresholds. These two analytical approaches are separately depicted in the figure: continuous models (blue), in which LASr was analyzed as a continuous variable, and categorical models (red), based on predefined LASr threshold values. Importantly, the effect estimates displayed in the figure derive from heterogeneous statistical models, including both hazard ratios and odds ratios, as well as different endpoint definitions and adjustment strategies across studies. Therefore, the plotted estimates should be interpreted as a qualitative visual representation of the overall direction and consistency of prognostic associations rather than as directly comparable quantitative measures. Despite methodological differences, both approaches consistently identify reduced atrial reservoir function as a marker of unfavorable prognosis.

**Figure 5:**
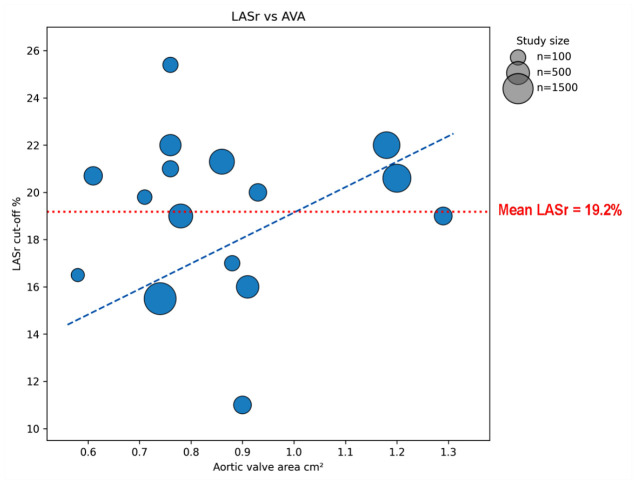
Scatter plot exploring the association between LASr cut-off values and AVA across studies. Each point represents an individual study, with bubble size proportional to sample size. The horizontal dashed line indicates the mean LASr cut-off value, while the regression line illustrates the overall trend between LASr thresholds and disease severity. The figure is intended as an exploratory descriptive representation and should not be interpreted as evidence of a definitive severity-threshold relationship between AVA and LASr. AVA, aortic valve area; LASr, left atrial reservoir strain.

A complementary perspective is provided by Figure 5, which explores the relationship between LASr cut-off values and AVA across the included studies.

The scatterplot suggests a trend toward lower LASr thresholds in patients with more severe aortic stenosis, as reflected by smaller valve areas. However, a certain degree of dispersion is evident, indicating variability in cut-off selection across studies.

Notably, most LASr cut-off values cluster within a relatively narrow range, despite differences in disease severity and study populations. The presence of a horizontal reference line highlights the overall central tendency of LASr thresholds, supporting the concept of a clinically relevant range for risk stratification. At the same time, the regression trend suggests a potential association between worsening valvular obstruction and progressive impairment of atrial reservoir function.

Bubble size further reflects study sample size, emphasizing that larger cohorts contribute substantially to the observed distribution, while smaller studies account for part of the variability.

The scatterplot suggests a possible exploratory trend toward lower LASr thresholds in patients with more severe AS, as reflected by smaller valve areas. However, a certain degree of dispersion is evident, indicating variability in cut-off selection across studies.

Notably, most LASr cut-off values cluster within a relatively narrow range, despite differences in disease severity and study populations. The presence of a horizontal reference line highlights the overall central tendency of LASr thresholds, which may support the hypothesis of a potentially clinically relevant reference range for risk stratification, although this observation should be interpreted cautiously given the heterogeneity of the included studies. At the same time, the regression trend suggests a possible association between worsening valvular obstruction and progressive impairment of atrial reservoir function. Bubble size further reflects study sample size, emphasizing that larger cohorts contribute substantially to the observed distribution, while smaller studies account for part of the variability.

Collectively, these findings support the role of LASr as a consistently reported predictor of adverse outcomes across a wide spectrum of patients with AS, with generally consistent associations across different study designs, populations, and endpoint definitions.

### 3.7. Methodological Quality and Risk of Bias Assessment

**Figure 6:**
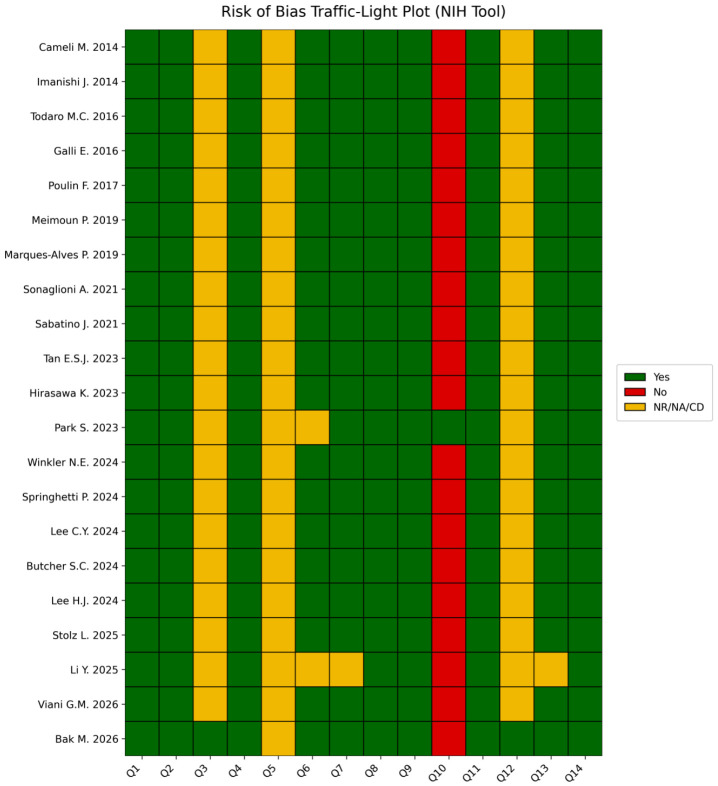
Traffic-light plot illustrating the risk of bias across individual studies [24,25,26,27,28,29,30,31,32,33,34,35,36,37,38,39,40,41,42,43,44] based on the NIH Quality Assessment Tool. Each row represents a single study, while each column corresponds to one of the 14 methodological domains (Q1–Q14). Color coding reflects the level of methodological quality: green indicates low risk of bias (“Yes”), red indicates high risk (“No”), and yellow denotes unclear or insufficient reporting (“NR/NA/CD”). NIH, National Institutes of Health; Q, quality assessment domain; Y, yes; N, no; NR/NA/CD, not reported/not applicable/cannot determine.

**Figure 7:**
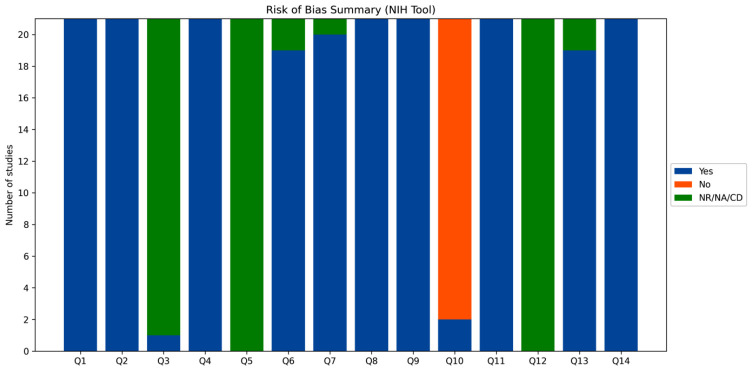
Bar chart summarizing the distribution of risk of bias ratings across all included studies [24,25,26,27,28,29,30,31,32,33,34,35,36,37,38,39,40,41,42,43,44] for each NIH assessment domain (Q1–Q14). Bars represent the number of studies classified as “Yes,” “No,” or “NR/NA/CD” for each criterion. NIH, National Institutes of Health; Q, quality assessment domain; Y, yes; N, no; NR/NA/CD, not reported/not applicable/cannot determine.

The methodological quality of the included studies is summarized in Table 6, with visual representations provided in Figure 6 and Figure 7.

Based on the NIH Quality Assessment Tool for observational cohort and cross-sectional studies, the overall methodological quality was rated as good in the majority of studies, with a limited number classified as fair.

Across domains, most studies consistently met key methodological criteria, including clearly defined research objectives, well-characterized study populations, and appropriate assessment of exposures and outcomes. These findings indicate an overall moderate risk of bias, with generally robust performance in core methodological aspects rather than uniformly low risk.

However, several recurrent limitations were identified. In particular, sample size justification and power calculations were rarely reported, representing one of the most consistent methodological gaps. Additionally, blinding of outcome assessors was frequently unclear or not described, potentially introducing detection bias.

The graphical summaries further highlight these patterns. The traffic-light plot (Figure 6) shows that most domains were rated as “yes,” although selected items—particularly those related to statistical power and blinding—display a higher proportion of “not reported” or “no” responses. The corresponding summary plot (Figure 7) confirms this distribution, with consistently high adherence across most domains but notable variability in a limited subset of criteria.

Moreover, heterogeneity in study design, follow-up duration, and endpoint definitions may represent additional sources of bias and limit direct comparability across studies. These aspects, while inherent to observational research, should be considered when interpreting pooled evidence.

Despite these limitations, the overall methodological profile was relatively consistent across studies, supporting the internal validity of the findings. Nonetheless, the presence of specific methodological weaknesses warrants cautious interpretation, particularly when generalizing results across different clinical settings.

## 4. Discussion

### 4.1. Main Findings

This systematic review provides a comprehensive and systematic synthesis of the available evidence regarding the prognostic role of LASr in patients with AS. Across a broad and heterogeneous body of literature, LASr consistently emerged as a powerful, reproducible, and clinically meaningful marker of adverse outcomes. Lower LASr values were robustly associated with an increased risk of mortality, heart failure hospitalization, and composite cardiovascular endpoints, with consistent findings across different analytical approaches, whether LASr was modeled as a continuous variable or categorized using study-specific cut-off thresholds.

In addition to its strong association with outcomes, LASr demonstrated clear and consistent incremental prognostic value beyond conventional echocardiographic parameters, including LVEF, GLS, and indices of diastolic dysfunction. Importantly, the persistence of its independent predictive value after multivariable adjustment underscores its ability to capture pathophysiological dimensions of cardiac dysfunction that are not adequately reflected by traditional measures, reinforcing its potential role as an integrative marker of disease burden.

Although the included studies were heterogeneous in terms of design, population characteristics, and clinical settings, the relative consistency in reported LASr cut-off values—generally clustering within a narrow range—suggests the presence of clinically relevant and potentially actionable thresholds, supporting its applicability in routine practice and risk stratification frameworks. Nevertheless, these findings should be interpreted in the context of the substantial inter-study heterogeneity observed across the included investigations, including differences in patient selection, AS severity, imaging acquisition protocols, software vendors, endpoint definitions, follow-up duration, and statistical adjustment strategies. Such methodological variability may have influenced the magnitude of reported prognostic associations and limits the direct comparability of effect estimates across studies.

A key finding of this review is the reproducibility of the prognostic significance of LASr across different imaging modalities, including both STE and CCT. This observation highlights the robustness and versatility of LASr as a biomarker of atrial function in AS, and supports its integration within a multimodality imaging approach. Notably, LASr values were systematically lower when assessed by CCT-based FT compared with echocardiography, likely reflecting methodological rather than biological differences, and emphasizing the need for modality-specific reference ranges when interpreting and applying cut-off thresholds in clinical practice.

### 4.2. Pathophysiological Mechanisms Underlying Left Atrial Reservoir Dysfunction in Aortic Stenosis

The impairment of LASr function in AS reflects the cumulative effects of chronic pressure overload on the left heart and the pulmonary circulation [46]. Progressive narrowing of the aortic valve increases LV afterload, promoting concentric hypertrophy and a gradual reduction in ventricular compliance [47]. As diastolic stiffness increases, LV filling pressures rise and are transmitted backward to the left atrium, exposing it to a sustained hemodynamic burden [48].

In response to this chronic load, the left atrium undergoes a process of structural and functional remodeling characterized by progressive dilatation, myocardial fibrosis, and loss of compliance [49]. These changes directly affect the reservoir phase of atrial function, during which the left atrium stores pulmonary venous return while the left ventricle is in systole. As atrial compliance declines, its ability to accommodate incoming blood is reduced, leading to a decrease in LASr [50].

Importantly, LASr provides a dynamic and integrative measure of atrial function that reflects not only intrinsic atrial properties but also upstream ventricular and valvular abnormalities [51]. In contrast to conventional volumetric indices, which primarily capture structural remodeling, LASr is sensitive to early functional impairment and may detect subtle alterations before overt atrial enlargement becomes apparent [52]. This makes it particularly valuable in the context of AS, where LVEF is often preserved until advanced stages of the disease [53].

Beyond its role as a marker of atrial remodeling, reduced LASr may also have direct pathophysiological consequences. Impaired reservoir function can limit ventricular filling during early diastole, contribute to reduced cardiac output reserve, and increase susceptibility to atrial arrhythmias [54]. The association between decreased LASr and atrial fibrillation observed in several studies supports the link between mechanical dysfunction and electrical instability [55].

Moreover, the reduction in LASr reflects the integrated impact of multiple pathophysiological pathways, including elevated filling pressures, ventricular–atrial coupling, and pulmonary vascular involvement [56]. As AS progresses, these interconnected mechanisms lead to a progressive decline in atrial performance, positioning LASr as a sensitive indicator of global cardiac decompensation [57].

Overall, left atrial reservoir dysfunction can be interpreted as an early and comprehensive marker of the hemodynamic burden imposed by AS, capturing the interplay between ventricular dysfunction, atrial remodeling, and pulmonary circulation involvement.

### 4.3. Clinical Implications for Risk Stratification and Patient Management

The findings of this systematic review suggest that LASr may represent a valuable tool for refining risk stratification in patients with AS, particularly in clinical scenarios where conventional parameters provide limited guidance.

In current practice, the timing of intervention is largely driven by symptom onset and indices of valvular severity, yet these criteria may not fully reflect the underlying burden of cardiac dysfunction [58]. The consistent association between reduced LASr and adverse outcomes highlights its potential role in identifying patients at higher risk, even in the absence of overt symptoms or marked impairment of LV systolic function. In this context, LASr may contribute to the detection of early stages of decompensation, when therapeutic intervention could still prevent irreversible myocardial damage.

The relative consistency of LASr thresholds across studies further supports its potential integration into routine clinical assessment. Although standardization is still required, the identification of clinically meaningful cut-off values may facilitate its use as an adjunctive parameter in decision-making algorithms, particularly in patients with borderline or discordant findings. However, the practical implementation of standardized LASr thresholds in routine clinical practice remains challenging due to the heterogeneity of imaging modalities, acquisition protocols, software vendors, and strain analysis methodologies currently used across centers. Consequently, currently proposed cut-off values should be interpreted cautiously and within the context of the specific imaging platform and analytical software employed.

Moreover, LASr may be especially informative in complex clinical settings, such as patients with preserved ejection fraction, low-flow low-gradient AS, or inconclusive symptom status [59,60]. In these conditions, where traditional markers may underestimate disease severity or functional impairment, atrial strain could provide additional insight into the hemodynamic impact of the disease [61].

Based on the cut-off values reported across the included studies, a pragmatic LASr threshold of approximately 19.2% may be considered as an exploratory and hypothesis-generating reference value for identifying patients with AS at increased risk of adverse events. In this framework, LASr should not be interpreted as a stand-alone indication for intervention, but rather as an adjunctive marker of cardiac damage to be integrated with symptoms, AS severity, LV function, diastolic parameters, biomarkers, and comorbidity burden. Patients with preserved LASr (>19.2%) may generally continue guideline-based surveillance, whereas those with reduced LASr (≤19.2%) may warrant closer clinical attention and more comprehensive multiparametric reassessment, particularly when additional high-risk features are present. This proposed approach should be interpreted cautiously, as it reflects a conceptual framework derived from currently available observational evidence rather than a validated evidence-based algorithm for routine clinical implementation.

In selected patients—particularly those with severe or discordant AS, preserved LVEF, uncertain symptom status, elevated natriuretic peptides, impaired LV-GLS, increased filling pressures, or progressive atrial remodeling—reduced LASr may support earlier consideration of aortic valve intervention before irreversible myocardial or atrial damage occurs.

**Figure 8:**
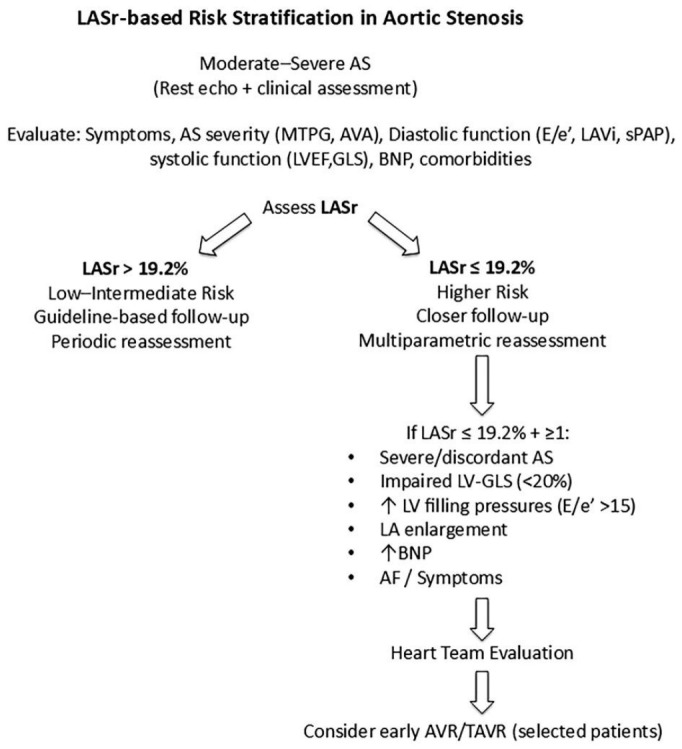
Proposed Practical Algorithm for LASr-Guided Risk Stratification in Aortic Stenosis. AS = aortic stenosis; LASr = left atrial reservoir strain; AVA = aortic valve area; MTPG = mean transvalvular pressure gradient; LVEF = left ventricular ejection fraction; GLS = global longitudinal strain; E/e′ = ratio of early mitral inflow velocity to mitral annular early diastolic velocity; LAVi = left atrial volume index; sPAP = systolic pulmonary artery pressure; BNP = B-type natriuretic peptide; AF = atrial fibrillation; AVR = aortic valve replacement; TAVR = transcatheter aortic valve replacement.

A proposed practical algorithm for the clinical use of LASr in AS is shown in Figure 8.

The incorporation of LASr into a multiparametric framework may further enhance its clinical utility. When interpreted alongside established variables—such as LV-GLS, diastolic function indices, and biomarkers—LASr may contribute to a more comprehensive and individualized assessment of patient risk.

Finally, the applicability of LASr across different imaging modalities, including both STE and CCT, expands its feasibility in contemporary clinical practice. This multimodality approach may be particularly relevant in patients undergoing advanced imaging for procedural planning, allowing simultaneous assessment of valvular anatomy and atrial function.

Importantly, while 2D-STE represents the reference method for LASr assessment due to its high temporal resolution and widespread availability, CCT with FT has emerged as a reliable complementary technique. Evidence from studies including patients with severe AS undergoing pre-procedural evaluation demonstrates a strong correlation between CCT-derived and echocardiographic measurements of LASr (r ≈ 0.79, *p* < 0.001), with only a modest systematic underestimation by CCT (mean bias approximately −3.7%) [62]. Absolute LASr values tend to be slightly lower when measured by CCT compared with echocardiography, likely reflecting intrinsic differences in temporal resolution and tracking algorithms. Importantly, agreement between modalities remains preserved across different rhythm conditions, although it may be somewhat attenuated in the presence of atrial fibrillation. These findings support the feasibility and reproducibility of CCT with FT for atrial strain assessment and highlight its potential role as an adjunctive modality, particularly in patients already undergoing CCT for anatomical evaluation. However, methodological differences between modalities, including frame rate, segmentation strategies, and vendor-specific software, currently limit direct interchangeability and emphasize the need for modality-specific reference ranges and further standardization. Specifically, inter-vendor variability in strain analysis software may further influence LASr measurements and the interpretation of proposed prognostic cut-off values. Differences in tracking algorithms, post-processing techniques, image quality optimization, and strain calculation methods across software platforms may contribute to variability in absolute LASr values and partially explain the heterogeneity observed among studies. Consequently, LASr thresholds should currently be interpreted within the context of the specific imaging modality and software platform used, further underscoring the importance of future standardization and vendor-independent validation studies.

### 4.4. Study Heterogeneity, Methodological Considerations, and Limitations

The interpretation of the findings of this systematic review should take into account several sources of heterogeneity across the included studies. The analyzed cohorts differed substantially in terms of clinical setting, encompassing asymptomatic patients, individuals with advanced disease, and populations undergoing surgical or transcatheter aortic valve interventions. This variability reflects real-world practice but may influence the generalizability of the results.

Methodological differences also represent an important source of variability. Although LASr was consistently evaluated, the techniques used for its assessment were not uniform, including both 2D-STE and CCT-based FT. In addition, vendor-specific software and differences in image acquisition and post-processing protocols may have contributed to variability in absolute LASr values and proposed cut-off thresholds.

An additional limitation relates to the intrinsic constraints of STE. The reproducibility of strain measurements may be influenced by several technical and operator-dependent factors, including variability between different software vendors [63], the level of operator expertise [64], and the quality of the acquired echocardiographic images. Furthermore, acquisition parameters such as frame rate [65], as well as hemodynamic conditions (e.g., loading status), may affect strain quantification. External mechanical influences, including chest wall configuration or deformities, may also interfere with image tracking and measurement accuracy [66].

Further heterogeneity arises from differences in endpoint definitions and follow-up duration. The included studies reported a wide range of clinical outcomes, including mortality, heart failure hospitalization, arrhythmic events, and composite endpoints, limiting direct comparability across studies. Similarly, the duration of follow-up varied considerably, potentially influencing the observed incidence of events and the strength of associations.

The observational design of the included studies represents an inherent limitation, with the potential for residual confounding and selection bias. Although the overall methodological quality was generally good, certain domains—such as sample size justification, blinding of outcome assessment, and completeness of reporting—were inconsistently addressed, as reflected in the quality assessment. In addition, several clinically relevant subgroup analyses could not be systematically explored because of inconsistent or insufficient reporting across the included studies. Specifically, data regarding sex-related differences, varying degrees of heart failure severity, chronic kidney disease, hepatic congestion, concomitant valvular disease, atrial fibrillation burden, and use or intensification of guideline-directed medical therapy were not uniformly available. Consequently, the potential influence of these factors on absolute LASr values and prognostic performance could not be reliably assessed within the present review.

Another limitation relates to the lack of standardized LASr cut-off values and the coexistence of different analytical approaches, including both continuous and categorical modeling. While this reflects the evolving nature of the field, it may limit the immediate clinical applicability of specific thresholds.

Despite these limitations, several strengths of the present review should be acknowledged. The analysis includes a large and diverse population, integrates data from multiple imaging modalities, and provides a comprehensive synthesis of prognostic evidence across different clinical scenarios. Importantly, the consistency in the direction of associations between reduced LASr and adverse outcomes across studies supports the robustness of the overall findings.

Overall, while heterogeneity and methodological differences should be considered when interpreting the results, the available evidence consistently supports the prognostic relevance of LASr in AS.

### 4.5. Future Perspectives

Future research should focus on consolidating the role of LASr within clinical decision-making pathways in AS. Although the current evidence consistently supports its prognostic relevance, prospective and multicenter studies are needed to validate standardized thresholds and to determine its incremental value when integrated into existing risk stratification models.

Particular attention should be directed toward the potential role of LASr in guiding the timing of intervention, especially in asymptomatic patients or in those with discordant grading of AS. In these settings, the identification of early functional impairment through atrial deformation imaging may help refine clinical decision-making and optimize patient outcomes.

From a methodological perspective, further efforts toward standardization of acquisition protocols, analysis techniques, and reporting of LASr are essential to improve reproducibility and facilitate widespread clinical adoption. The development of vendor-independent software and consensus recommendations may represent important steps in this direction [67].

The integration of LASr into multimodality imaging frameworks also represents a promising area of investigation. The increasing use of CCT for procedural planning offers the opportunity to incorporate atrial functional assessment into routine workflows, potentially enhancing the overall evaluation of patients with AS.

In addition, emerging technologies, including artificial intelligence-based image analysis, may further improve the accuracy, reproducibility, and scalability of LASr measurements, enabling their implementation in large-scale clinical settings.

Finally, longitudinal studies evaluating temporal changes in LASr and their relationship with disease progression and response to therapeutic interventions are warranted. Such investigations may provide valuable insights into the dynamic nature of atrial remodeling and its potential role as a therapeutic target in AS.

## 5. Conclusions

This systematic review supports the prognostic relevance of LASr in patients with AS. Across different clinical settings and imaging modalities, reduced LASr was consistently associated with adverse outcomes, including mortality, heart failure hospitalization, arrhythmic events, and composite cardiovascular endpoints.

LASr appears to provide information beyond conventional measures of AS severity and LV function, reflecting the cumulative burden of left-sided pressure overload, diastolic dysfunction, and atrial remodeling. Its relatively consistent prognostic thresholds across studies suggest a potentially clinically relevant reference range for refined risk stratification, particularly in patients with preserved ejection fraction, uncertain symptom status, or discordant disease severity. However, these threshold values derive from heterogeneous observational studies using different imaging modalities, vendor-specific software platforms, post-processing methodologies, patient populations, and endpoint definitions, and therefore should be interpreted cautiously. In particular, the pragmatic LASr threshold of approximately 19.2% should be considered hypothesis-generating rather than definitively clinically actionable, pending further prospective validation and methodological standardization.

Although further prospective multicenter studies are needed to standardize measurement protocols, validate clinically actionable cut-off values, and define its role in therapeutic decision-making, LASr represents a promising and clinically meaningful marker of cardiac damage in AS. Its integration into multiparametric and multimodality assessment may improve identification of high-risk patients and support more individualized management strategies.

## Figures and Tables

**Table 1:** Main Characteristics of the Included Studies [24,25,26,27,28,29,30,31,32,33,34,35,36,37,38,39,40,41,42,43,44] Evaluating Left Atrial Reservoir Strain in Aortic Stenosis.

Study (Year), Country	Design	Method	Software	Size (% Males)	Age (yrs)	AVA (cm^2^)	Prognostic Cut-Off
Cameli M. (2014), Italy [24]	Prospective, single-center	2D-STE	GE	76 (68.2)	67.5	NR	LASr 16.9%
Imanishi J. (2014), Japan [25]	Retrospective, single-center	2D-STE	Toshiba	27 (30)	76	0.61	LA end-diastolic SR < 0.79 s^−1^
Todaro M.C. (2016), Italy [26]	Prospective, single-center	2D-STE	GE	82 (37.8)	73	0.71	LASr 19.8%
Galli E. (2016), France [27]	Retrospective, single-center	2D-STE	GE	128 (57)	78.9	0.76	LASr 21%
Poulin F. (2017), Canada [28]	Retrospective, single-center	2D-STE	Siemens	52 (54)	81	0.70	NR
Meimoun P. (2019), France [29]	Prospective, single-center	2D-STE	GE	102 (50)	77	0.88	LASr 17%
Marques-Alves P. (2019), Portugal [30]	Retrospective, single-center	2D-STE	GE	182 (51)	76	0.90	LASr 11%
Sonaglioni A. (2021), Italy [31]	Retrospective, single-center	2D-STE	Philips	186 (61.8)	71.9	1.29	LASr 19%
Sabatino J. (2021), Italy [32]	Prospective, single-center	2D-STE	GE	100 (48)	81.2	0.75	NR
Tan E.S.J. (2023), Singapore [33]	Prospective, multicenter	2D-STE	GE	173 (54.9)	69.1	0.93	LASr 20%
Hirasawa K. (2023), Netherlands [34]	Retrospective, single-center	2D-TTE + CT-FT	GE + Medis	376 (53)	80	0.76	LASr 22%
Park S. (2023), Korea [35]	Prospective, multicenter	CT-FT	Medis	211 (41.7)	81	0.61	LASr 20.7%
Winkler N.E. (2024), Switzerland [36]	Retrospective, single-center	2D-STE	TomTec	198 (53.5)	81	0.80	LASct –6.8%
Springhetti P. (2024), Italy [37]	Retrospective, multicenter	2D-STE	TomTec	467 (50.7)	80.6	0.91	LASr 16%
Lee C.Y. (2024), Taiwan [38]	Retrospective, single-center	2D-STE	TomTec	712 (47)	78	0.86	LASr 21.3%
Butcher S.C. (2024), Netherlands [39]	Retrospective, single-center	2D-STE	GE	601 (53)	81	0.78	LASr 19%
Lee H.J. (2024), Korea [40]	Retrospective, single-center	2D-STE	TomTec	923 (54.9)	74	1.18	LASr 22%
Stolz L. (2025), Germany [41]	Retrospective, single-center	2D-STE	TomTec	1888 (55.7)	81	0.74	LASr 15.5%
Li Y. (2025), China [42]	Prospective, single-center	2D-STE	GE	56 (55.4)	68.7	0.58	LASr 16.5%
Viani G.M. (2026), Switzerland [43]	Retrospective, single-center	2D-STE	Philips	99 (51.2)	81.6	0.76	LASr 25.4%
Bak M. (2026), Korea [44]	Retrospective, single-center	2D-STE	TomTec	1125 (52.8)	74	1.20	LASr 20.6%

Data are presented as reported in the original studies. Study design, imaging modality, software used for strain analysis, sample size, demographic characteristics, aortic valve area, and proposed prognostic cut-off values for left atrial reservoir strain are summarized for each study. 2D, two-dimensional; AVA, aortic valve area; CT-FT, computed tomography feature tracking; GE, General Electric; LA, left atrial; LASr, left atrial reservoir strain; LASct, left atrial strain by computed tomography; NR, not reported; SR, strain rate; STE, speckle-tracking echocardiography; TTE, transthoracic echocardiography.

**Table 2:** Baseline Clinical Profile, Comorbidities, and Medical Therapy of the Study Population.

Parameter	Weighted Median	Weighted IQR (Q1–Q3)	Studies Included	N Patients
**Age (years)**	80	74–81	21	7764
**Males (%)**	54	51–56	21	7764
**BSA (m^2^)**	1.80	1.70–1.85	18	7455
**BMI (kg/m^2^)**	25.5	24.2–26.6	11	5720
**HR (bpm)**	74	72–77	6	1139
**SBP (mmHg)**	132	121.8–139	7	2126
**DBP (mmHg)**	72	67–75	6	2069
**Hypertension (%)**	65	61.5–75	17	7038
**Diabetes (%)**	28	27–33	18	7226
**Smoking (%)**	25.5	16–37.6	9	2363
**Dyslipidemia (%)**	57	43–64	13	5172
**CAD (%)**	32	18–59	16	6678
**Prior stroke/CVD (%)**	9	2.8–18	9	3762
**NYHA ≥ 3 (%)**	31	11–58	12	5499
**AF history (%)**	16	6.8–27	10	4694
**HF at diagnosis (%)**	23	14–26	5	1787
**Previous HF (%)**	15.7	5.2–17.5	4	1663
**PVD (%)**	2	0.4–12	6	2140
**COPD (%)**	15	7.8–23	7	2823
**CKD (%)**	28.5	24–37	5	2756
**eGFR (mL/min)**	61	54–64	7	3721
**Dialysis (%)**	3	2.6–6.1	3	2087
**NT-proBNP (pg/mL)**	312	180–1006	7	2815
**Antiplatelets (%)**	67	33–67	2	213
**Anticoagulants (%)**	6.5	—	1	186
**ACEi/ARBs (%)**	53	49–54	7	2574
**CCB (%)**	27.9	12.2–63	3	295
**BB (%)**	48	31.8–58	6	2489
**Diuretics (%)**	54	29.6–56	5	1367
**MRA (%)**	14	11–14	2	1152
**Statins (%)**	46	28.5–63	4	1988

Data are expressed as weighted medians with corresponding interquartile ranges (IQR; Q1–Q3), calculated according to the sample size of each study. The column “Studies included” indicates the number of studies contributing data for each variable, whereas “N patients” refers to the total number of individuals included in the analysis for that specific parameter. ACEi, angiotensin-converting enzyme inhibitors; ARBs, angiotensin II receptor blockers; AF, atrial fibrillation; BB, beta-blockers; BMI, body mass index; BSA, body surface area; CAD, coronary artery disease; CCB, calcium channel blockers; CKD, chronic kidney disease; COPD, chronic obstructive pulmonary disease; CVD, cerebrovascular disease; DBP, diastolic blood pressure; eGFR, estimated glomerular filtration rate; HF, heart failure; HR, heart rate; IQR, interquartile range; MRA, mineralocorticoid receptor antagonists; NT-proBNP, N-terminal pro–B-type natriuretic peptide; NYHA, New York Heart Association; PVD, peripheral vascular disease; Q1, first quartile; Q3, third quartile; SBP, systolic blood pressure.

**Table 3:** Baseline Transthoracic Echocardiographic Parameters of the Study Population.

Parameter	Weighted Median	Weighted IQR (Q1–Q3)	Studies Included	N Patients
**IVS (mm)**	12.8	12.0–12.9	4	536
**PW (mm)**	11.9	—	1	56
**LVEDD (mm)**	49	47–51.6	9	2964
**LVESD (mm)**	31	29–33.8	7	2713
**RWT**	0.46	0.44–0.51	4	1603
**LVMi (g/m^2^)**	119	108–126	13	5786
**LVEDV (m** **L** **)**	113.6	101.5–144.1	7	2487
**LVESV (m** **L** **)**	50	37.5–72.4	7	2487
**LVEF (%)**	58	55–63	21	7764
**SV (m** **L** **)**	70	68–81	11	3411
**E/A**	0.83	0.73–1.02	11	2820
**E/e′**	15	13.2–17	17	5716
**Peak transaortic gradient (mmHg)**	65	51.8–85.5	9	3247
**Mean transaortic gradient (mmHg)**	40	32–50	17	6506
**AVA (cm^2^)**	0.78	0.70–0.91	20	7688
**LA diameter (mm)**	42	35.1–42	2	979
**LAVi (m** **L** **/m^2^)**	43	40–48	21	7764
**>mild AR (%)**	4	0–11	7	3701
**>mild MR (%)**	3	0–20	8	3876
**>mild TR (%)**	16	3.7–16	4	3167
**TAPSE (mm)**	21	18–22	7	1447
**sPAP (mmHg)**	35	30–39	12	4474

Data are expressed as weighted medians with corresponding interquartile ranges (IQR; Q1–Q3), calculated according to the sample size of each study. The column “Studies included” indicates the number of studies contributing data for each variable, whereas “N patients” refers to the total number of individuals included for that specific parameter. AR, aortic regurgitation; AVA, aortic valve area; E/A, ratio of early to late diastolic transmitral flow velocity; E/e′, ratio of early transmitral flow velocity to early diastolic mitral annular velocity; IQR, interquartile range; IVS, interventricular septum; LA, left atrial; LAVi, left atrial volume index; LVEDD, left ventricular end-diastolic diameter; LVEDV, left ventricular end-diastolic volume; LVEF, left ventricular ejection fraction; LVESD, left ventricular end-systolic diameter; LVESV, left ventricular end-systolic volume; LVMi, left ventricular mass index; MR, mitral regurgitation; PW, posterior wall; Q1, first quartile; Q3, third quartile; RWT, relative wall thickness; sPAP, systolic pulmonary artery pressure; SV, stroke volume; TAPSE, tricuspid annular plane systolic excursion; TR, tricuspid regurgitation.

**Table 4:** Left Atrial Strain and Myocardial Deformation Parameters of the Study Population.

Parameter	Weighted Median	Weighted IQR (Q1–Q3)	Studies Included	N Patients
**LAScd (%)**	11.7	10.8–18.7	12	4916
**LASct (%)**	10.0	9.0–14.2	11	4806
**LASr (%)**	22.1	17–29.5	20	7737
**LA systolic SR (s^−1^)**	0.89	0.89–1.35	4	447
**LA early diastolic SR (s^−1^)**	0.70	0.51–0.80	5	529
**LA end-diastolic SR (s^−1^)**	1.10	0.93–1.30	5	529
**E/e′/LASr**	0.90	—	1	82
**LV-GLS (%)**	15.4	14.3–17.6	13	5344
**LV-GCS (%)**	21.5	—	1	82
**LV-GRS (%)**	43	—	1	82
**RAScd (%)**	15.2	—	1	1888
**RASct (%)**	6.6	—	1	1888
**RASr (%)**	21.6	—	1	1888

Data are expressed as weighted medians with corresponding interquartile ranges (IQR; Q1–Q3), calculated according to the sample size of each study. The column “Studies included” indicates the number of studies contributing data for each variable, whereas “N patients” refers to the total number of individuals included in the analysis for that specific parameter. E/e’, ratio of early transmitral flow velocity to early diastolic mitral annular velocity; IQR, interquartile range; LA, left atrial; LAScd, left atrial conduit strain; LASct, left atrial contractile strain; LASr, left atrial reservoir strain; LV, left ventricular; LV-GCS, left ventricular global circumferential strain; LV-GLS, left ventricular global longitudinal strain; LV-GRS, left ventricular global radial strain; Q1, first quartile; Q3, third quartile; RAScd, right atrial conduit strain; RASct, right atrial contractile strain; RASr, right atrial reservoir strain; SR, strain rate.

**Table 5:** Follow-up Duration, Clinical Outcomes, and Prognostic Predictors Across Included Studies [24,25,26,27,28,29,30,31,32,33,34,35,36,37,38,39,40,41,42,43,44].

Study	Follow-Up (Months)	Events n (%)	Endpoint	Main Predictors	Cut-Off
Cameli M. [24]	0.06	15 (19.7)	Post-operative AF	LASr (HR 0.96)	LASr 16.9%
Imanishi J. [25]	12	15 (56)	New-onset post-op AF	LA end-diastolic SR (HR 0.0052)	<0.79 s^−1^
Todaro M.C. [26]	16	53 (64.6)	Symptoms + death	LV-GLS (HR 1.49), LASr (HR 0.87)	LASr 19.8%
Galli E. [27]	14	38 (29.7)	Death + HF + hospitalization	LASr < 21% (HR 2.88), CAD (HR 2.68), NYHA > 2 (HR 2.08)	LASr 21%
Poulin F. [28]	N/A	N/A	Post-TAVI AF	LA early diastolic SR (OR 1.8 per 0.10 s^−1^)	NR
Meimoun P. [29]	25	53 (52)	Death + HF hospitalization	LASr (HR 0.95), Charlson score (HR 1.38)	LASr 17%
Marques-Alves P. [30]	30	NR	HF + death + AVR	LASr (HR 0.90)	LASr 11%
Sonaglioni A. [31]	27.6	63 (33.9)	CV hosp + AVR/TAVR + death	DM (HR 1.87), NT-proBNP (HR 1.14), E/e’ (HR 1.07), LASr (HR 0.85)	LASr 19%
Sabatino J. [32]	31	35 (35)	CV death + HF hospitalization	ΔLAS (HR 0.80)	NR
Tan E.S.J. [33]	32	66 (38)	Death + HF + progression	LAScd < 6% (HR 3.08), LASr < 20% (HR 1.92)	LASr 20%
Hirasawa K. [34]	45	148 (39)	All-cause mortality	LASr (HR 0.96)	LASr 22%
Park S. [35]	6	52 (24.6)	Post-TAVR arrhythmia	Peak velocity (OR 1.78), LA volume (OR 1.01), LV-GLS, LASr	LASr 20.7%
Winkler N.E. [36]	60	49 (24.7)	CV mortality	LASct (HR 1.10), age (HR 1.08)	LASct −6.8%
Springhetti P. [37]	19.2	96 (20.5)	Death + HF hospitalization	LASr (HR 0.95), age (HR 1.04)	LASr 16%
Lee C.Y. [38]	18	93 (13.1)	Death + MACE	LASr (HR 0.97)	LASr 21.3%
Butcher S.C. [39]	40	258 (43)	All-cause death	LASr < 19% (HR 1.28)	LASr 19%
Lee H.J. [40]	70.8	186 (20.2)	Death + HF hospitalization	LASr (HR 0.97)	LASr 22%
Stolz L. [41]	36	556 (29.5)	All-cause mortality	LASr/RASr (HR 1.72), combined (HR 2.45)	LASr 15.5%
Li Y. [42]	N/A	N/A	Advanced HF symptoms	LASr (OR 0.58), LAEI (OR 0.92)	LASr 16.5%
Viani G.M. [43]	24	43 (43.4)	Death + MI + PPM + stroke	LAScd (AUC 0.93)	LASr 25.4%
Bak M. [44]	42.8	381 (33.9)	Cardiac death + HF hospitalization	LASr (HR 0.94)	LASr 20.6%

Data are reported as provided in the original studies. Follow-up duration, event rates, endpoint definitions, main prognostic predictors, and corresponding cut-off values are summarized for each study. Hazard ratios (HR) and odds ratios (OR) are reported when available. The column “Events” indicates the number and percentage of patients experiencing the outcome of interest. AF, atrial fibrillation; AVR, aortic valve replacement; CAD, coronary artery disease; CV, cardiovascular; DM, diabetes mellitus; HF, heart failure; HR, hazard ratio; LA, left atrial; LAEI, left atrial expansion index; LAScd, left atrial conduit strain; LASct, left atrial contractile strain; LASr, left atrial reservoir strain; LV, left ventricular; LV-GLS, left ventricular global longitudinal strain; MACE, major adverse cardiovascular events; MI, myocardial infarction; NR, not reported; NT-proBNP, N-terminal pro–B-type natriuretic peptide; OR, odds ratio; PPM, permanent pacemaker implantation; SR, strain rate; TAVI, transcatheter aortic valve implantation.

**Table 6:** Methodological Quality Assessment of Included Studies [24,25,26,27,28,29,30,31,32,33,34,35,36,37,38,39,40,41,42,43,44] Using the NIH Tool.

Study	Q1	Q2	Q3	Q4	Q5	Q6	Q7	Q8	Q9	Q10	Q11	Q12	Q13	Q14	Overall
Cameli M. [24]	Y	Y	NR	Y	NR	Y	Y	Y	Y	N	Y	NR	Y	Y	Good
Imanishi J. [25]	Y	Y	NR	Y	NR	Y	Y	Y	Y	N	Y	NR	Y	Y	Good
Todaro M.C. [26]	Y	Y	NR	Y	NR	Y	Y	Y	Y	N	Y	NR	Y	Y	Good
Galli E. [27]	Y	Y	NR	Y	NR	Y	Y	Y	Y	N	Y	NR	Y	Y	Good
Poulin F. [28]	Y	Y	NR	Y	NR	Y	Y	Y	Y	N	Y	NR	Y	Y	Good
Meimoun P. [29]	Y	Y	NR	Y	NR	Y	Y	Y	Y	N	Y	NR	Y	Y	Good
Marques-Alves P. [30]	Y	Y	NR	Y	NR	Y	Y	Y	Y	N	Y	NR	Y	Y	Good
Sonaglioni A. [31]	Y	Y	NR	Y	NR	Y	Y	Y	Y	N	Y	NR	Y	Y	Good
Sabatino J. [32]	Y	Y	NR	Y	NR	Y	Y	Y	Y	N	Y	NR	Y	Y	Good
Tan E.S.J. [33]	Y	Y	NR	Y	NR	Y	Y	Y	Y	N	Y	NR	Y	Y	Good
Hirasawa K. [34]	Y	Y	NR	Y	NR	Y	Y	Y	Y	N	Y	NR	Y	Y	Good
Park S. [35]	Y	Y	NR	Y	NR	CD	Y	Y	Y	Y	Y	NR	Y	Y	Fair
Winkler N.E. [36]	Y	Y	NR	Y	NR	Y	Y	Y	Y	N	Y	NR	Y	Y	Good
Springhetti P. [37]	Y	Y	NR	Y	NR	Y	Y	Y	Y	N	Y	NR	Y	Y	Good
Lee C.Y. [38]	Y	Y	NR	Y	NR	Y	Y	Y	Y	N	Y	NR	Y	Y	Good
Butcher S.C. [39]	Y	Y	NR	Y	NR	Y	Y	Y	Y	N	Y	NR	Y	Y	Good
Lee H.J. [40]	Y	Y	NR	Y	NR	Y	Y	Y	Y	N	Y	NR	Y	Y	Good
Stolz L. [41]	Y	Y	NR	Y	NR	Y	Y	Y	Y	N	Y	NR	Y	Y	Good
Li Y. [42]	Y	Y	NR	Y	NR	CD	NA	Y	Y	N	Y	NR	NA	Y	Fair
Viani G.M. [43]	Y	Y	NR	Y	NR	Y	Y	Y	Y	N	Y	NR	Y	Y	Good
Bak M. [44]	Y	Y	Y	Y	NR	Y	Y	Y	Y	N	Y	Y	Y	Y	Good

The methodological quality of the included studies was evaluated using the National Institutes of Health (NIH) Quality Assessment Tool for observational cohort and cross-sectional studies. Each study was assessed across 14 predefined domains (Q1–Q14), reflecting key aspects of internal validity. Individual items were rated as “Yes,” “No,” or “Not Reported/Not Applicable/Cannot Determine (NR/NA/CD),” and an overall quality rating (Good or Fair) was assigned accordingly. NIH, National Institutes of Health; Q, quality assessment domain; Y, yes; N, no; NR, not reported; NA, not applicable; CD, cannot determine.

## Data Availability

Data extracted from included studies will be publicly available on Zenodo (https://zenodo.org).

## Supplementary Materials

The following supporting information can be downloaded at https://www.mdpi.com/article/10.3390/jcm15114304/s1: Supplementary Materials File S1: PRISMA 2020 checklist; Supplementary Materials File S2: The full INPLASY record [1,2,3,4,5,6,7,8,9,10,11,12,13,14,15,16,17,18,19,20,21] (https://inplasy.com/inplasy-2026-4-0099/, accessed on 27 April 2026).
