# Supplementary material for: Prognostic Role of Left Atrial Reservoir Strain for Risk Stratification in Aortic Stenosis: A Systematic Review
Source: J Clin Med. 2026 Jun 2;15(11):4304. doi: 10.3390/jcm15114304 (PMC13258756; doi:10.3390/jcm15114304)
Supplement: Supplementary file 1 [file jcm-15-04304-s001.zip › Supplementary Materials File S2.pdf]

International Platform of Registered Systematic Review and Meta-analysis Protocols

INPLASY

Prognostic Role of Left Atrial Reservoir Strain for Risk Stratifi-cation in Aortic Stenosis: A Systematic Review

INPLASY202640099
doi: 10.37766/inplasy2026.4.0099
Received: 27 April 2026
Published: 27 April 2026

Sonaglioni, A; Baravelli, M; Gramaglia, GF; Nicolosi GL; Lombardo, M.

Corresponding author:
Andrea Sonaglioni

sonaglioniandrea@gmail.com

Author Affiliation:
IRCCS MultiMedica.

ADMINISTRATIVE INFORMATION

Support - Ministero della Salute Ricerca Corrente.

Review Stage at time of this submission - Completed but not published.

Conflicts of interest - None declared.

INPLASY registration number: INPLASY202640099

Amendments - This protocol was registered with the International Platform of Registered Systematic Review and Meta-Analysis Protocols (INPLASY) on 27 April 2026 and was last updated on 27 April 2026.

INTRODUCTION

Review question / Objective The present systematic review aims to evaluate the prognostic role of left atrial reservoir strain (LASr) in patients with aortic stenosis (AS), integrating findings across different imaging modalities and clinical settings. In addition, this work seeks to characterize the clinical and echocardiographic profiles of the studied populations and to provide an overview of the main determinants of adverse outcomes, with the goal of better defining the role of LASr in contemporary risk stratification.

Rationale Over the past decade, several studies have explored the prognostic significance of LASr in patients with AS across different clinical scenarios, including asymptomatic individuals, patients undergoing transcatheter or surgical valve intervention, and those with varying degrees of ventricular function. These investigations have consistently suggested that reduced LASr is associated with an increased risk of adverse outcomes, including mortality, heart failure hospitalization, and arrhythmic events, as also summarized in recent review articles synthesizing the available prognostic evidence [14,15].

In addition to LASr, other imaging-derived parameters—such as left ventricular global longitudinal strain [16], indices of diastolic dysfunction [17], and markers of atrial enlargement [18]—have been proposed as complementary prognostic indicators. Clinical variables, including age [19], comorbidities [20], and biomarkers such as natriuretic peptides [21], further contribute to risk stratification, underscoring the multifactorial nature of disease progression in AS.

Nevertheless, the available evidence remains heterogeneous, with considerable variability in study design, imaging methodologies, LASr cut-off values, and endpoint definitions. This heterogeneity limits the direct comparability of individual studies and complicates the integration of LASr into routine clinical decision-making.

In light of these considerations, a comprehensive synthesis of the current evidence is warranted.

Sonaglioni et al. INPLASY protocol 202640099. doi:10.37766/inplasy2026.4.0099 Downloaded from https://inplasy.com/inplasy-2026-4-0099/

References
14. https://doi.org/10.1111/echo.15829.
15. https://doi.org/10.3389/fcvm.2025.1667871.
16. https://doi.org/10.1016/j.echo.2022.03.008.
17. https://doi.org/10.1136/heartjnl-2022-320886.
18. https://doi.org/10.1161/JAHA.117.006615.
19. https://doi.org/10.31128/AJGP-08-22-6527.
20.https://doi.org/10.1097/MD.0000000000036283.
21. https://doi.org/10.1136/heartjnl-2018-313746.

Condition being studied Aortic stenosis (AS) is the most common valvular heart disease in developed countries [1] and represents a major cause of cardiovascular morbidity and mortality, particularly in the aging population [2]. Its clinical course is characterized by a prolonged asymptomatic phase followed by rapid clinical deterioration once symptoms develop, making the identification of early markers of disease progression a central challenge in contemporary cardiology.
Current management strategies rely largely on symptom onset and conventional echocardiographic parameters, such as aortic valve area and transvalvular gradients, to guide the timing of intervention [3,4]. However, these indices primarily reflect valvular obstruction and do not fully capture the complex myocardial and hemodynamic consequences of the disease. As a result, patients may remain clinically stable despite ongoing subclinical cardiac remodeling, with potential implications for long-term outcomes [5].
Chronic pressure overload in AS induces a cascade of structural and functional changes involving not only the left ventricle but also the left atrium and the pulmonary circulation [6,7]. While left ventricular adaptation has been extensively studied, increasing attention has been directed toward the left atrium as an early and sensitive marker of cardiac dysfunction [8]. In this context, left atrial remodeling reflects the cumulative effects of elevated filling pressures, impaired diastolic function, and reduced ventricular compliance [9].
Left atrial reservoir strain (LASr), assessed through speckle-tracking echocardiography and, more recently, cardiac computed tomography, has emerged as a reproducible and quantitative measure of atrial function [10]. Unlike conventional volumetric parameters, LASr provides a direct assessment of atrial compliance and reservoir capacity, offering incremental insight into the hemodynamic burden imposed by AS [11]. Importantly, impairment of LASr may precede overt changes in left ventricular systolic function, making it a potential early marker of disease progression [12,13].

References
1. https://doi.org/10.3390/jcm12062178.
2. https://doi.org/10.3390/jcm14238276.
3. https://doi.org/10.5551/jat.RV22023.
4. https://doi.org/10.1016/j.acvd.2025.10.325.
5. https://doi.org/10.3390/jcm13051200.
6. https://doi.org/10.1001/jama.2024.16477.
7. https://doi.org/10.1016/j.acvd.2025.11.004.
8. https://doi.org/10.1002/ejhf.2562.
9. https://doi.org/10.1016/j.jacc.2019.01.059.
10. https://doi.org/10.31083/RCM46198.
11. https://doi.org/10.3390/medicina60050693.
12. https://doi.org/10.1186/s12872-022-02532-w.
13. https://doi.org/10.3390/jpm14111093.

METHODS

Search strategy A systematic and comprehensive literature search was independently carried out by two investigators to identify studies assessing the prognostic significance of LASr in patients with aortic stenosis. Studies including adult populations across the full spectrum of disease severity were considered eligible, in order to reflect real-world clinical heterogeneity.
Electronic databases, namely PubMed, Scopus, and EMBASE, were searched from their inception through March 2026. The search strategy integrated both controlled vocabulary and free-text terms related to aortic stenosis and atrial deformation imaging, including “aortic stenosis”, “left atrial strain”, “left atrial reservoir strain”, “speckle-tracking echocardiography”, “cardiac computed tomography”, “feature tracking”, “cardiac deformation”, “prognosis”, “clinical outcome”, and “risk prediction”. Studies employing different imaging modalities for LASr assessment, including echocardiography and cardiac computed tomography, were considered.
No restrictions were applied with regard to language, publication year, or geographic location. Furthermore, to ensure completeness of the evidence base, the reference lists of all selected articles and relevant review papers were manually examined to identify additional studies.
Any disagreements between reviewers during the screening and selection process were resolved by discussion and consensus, with adjudication by a third reviewer when necessary.

Participant or population Patients with moderate and severe aortic stenosis.

Intervention STE- and/or CT-derived LASr measurments in patients with moderate and severe aortic stenosis.

Comparator N/A.

Sonaglioni et al. INPLASY protocol 202640099. doi:10.37766/inplasy2026.4.0099 Downloaded from https://inplasy.com/inplasy-2026-4-0099/

Study designs to be included Observational Cohort and Cross-Sectional Studies.

Eligibility criteria Studies were considered eligible if they fulfilled predefined inclusion criteria. In particular, only observational studies, including both prospective and retrospective cohort designs, were included. Eligible studies were required to enroll adult patients with moderate or severe aortic stenosis and to assess LASr using validated imaging techniques, such as two-dimensional speckle-tracking echocardiography or cardiac computed tomography with feature-tracking analysis.

Furthermore, studies had to report clinical outcomes or prognostic endpoints, including all-cause mortality, cardiovascular mortality, heart failure hospitalization, arrhythmic events, or composite cardiovascular outcomes. Only studies providing extractable data on the association between LASr and clinical outcomes were considered.

To ensure methodological consistency, studies focusing exclusively on diagnostic performance without reporting prognostic data were excluded. Additional exclusion criteria included studies with mixed valvular populations without separate analysis of aortic stenosis, lack of clearly defined cohorts, or absence of relevant outcome data.

Case reports, editorials, conference abstracts, narrative reviews, and experimental or preclinical studies were not eligible for inclusion.

Information sources Electronic databases, namely PubMed, Scopus, and EMBASE, were searched from their inception through April 2026.

Main outcome(s) To assess the diagnostic and prognostic role of STE- and/or CT-derived LASr in patients with moderate and severe aortic stenosis.

Additional outcome(s) To compare STE- and/or CT-derived LASr values in patients with moderate and severe aortic stenosis.

Data management Study selection was performed independently by two reviewers. All retrieved records were initially screened based on title and abstract, followed by full-text evaluation of potentially eligible studies according to the predefined inclusion criteria. Disagreements were resolved by consensus, with arbitration by a third reviewer when necessary.

Data extraction was carried out using a standardized form developed prior to study initiation. Extracted information included study characteristics such as first author, year of publication, country, study design, imaging modality, and analysis software, as well as sample size and demographic features of the study population.

Clinical and baseline variables were systematically collected when available, including age, sex distribution, anthropometric parameters, cardiovascular risk factors, comorbidities, and ongoing medical therapy.

Echocardiographic and imaging data were extracted in detail, focusing on parameters of left ventricular structure and function, including ventricular dimensions, volumes, mass, and systolic performance assessed by left ventricular ejection fraction and global longitudinal strain. Diastolic function and filling pressures were evaluated using Doppler-derived indices, particularly the E/e' ratio. In addition, markers of aortic stenosis severity, such as aortic valve area and transvalvular pressure gradients, were systematically recorded.

Left atrial function was specifically assessed through myocardial deformation parameters, including left atrial reservoir, conduit, and contractile strain, as well as strain rate indices when available. Additional deformation measures, such as left ventricular strain and right atrial strain parameters, were also collected when reported.

Outcome data were extracted for all included studies, including duration of follow-up, definition of clinical endpoints, and the main prognostic predictors identified in each analysis. Particular attention was given to the association between LASr and adverse outcomes, including both continuous and categorical (cut-off–based) analyses.

All extracted data were independently verified by both reviewers, and any discrepancies were resolved through re-evaluation of the original articles.

Quality assessment / Risk of bias analysis The methodological quality and risk of bias of the included studies were independently assessed by two reviewers using the National Institutes of Health (NIH) Quality Assessment Tool for Observational Cohort and Cross-Sectional Studies. This instrument evaluates multiple domains of internal validity, including the clarity of the research question, definition of the study population, adequacy of exposure and outcome assessment, consistency of measurement methods, control of confounding factors, and appropriateness of statistical analyses. Each item was rated as "Yes", "No", or "Not Reported/Not Applicable", according to predefined criteria.

An overall quality rating (Good, Fair, or Poor) was assigned to each study based on the number of criteria fulfilled and the overall methodological

Sonaglioni et al. INPLASY protocol 202640099. doi:10.37766/inplasy2026.4.0099 Downloaded from https://inplasy.com/inplasy-2026-4-0099/

rigor. Discrepancies between reviewers were resolved through discussion and consensus.
The results of the quality assessment were summarized both descriptively and through graphical representations, including a traffic-light plot and a risk-of-bias summary, to provide a comprehensive overview of methodological quality across studies.

Strategy of data synthesis The substantial variability across studies in terms of design, imaging techniques (including both echocardiography and computed tomography), patient characteristics, and outcome definitions precluded the performance of a formal quantitative meta-analysis. Therefore, the available evidence was integrated through a structured qualitative and descriptive approach.
To summarize baseline characteristics, aggregate estimates were derived from study-level data. Continuous variables were expressed as weighted medians with corresponding interquartile ranges (IQR), applying weights proportional to the sample size of each study. When data were reported as mean and standard deviation, approximate distributions were inferred to ensure comparability across datasets.
This methodology enabled a harmonized synthesis of demographic, clinical, and imaging-derived variables, including measures of aortic stenosis severity, left ventricular function, and left atrial mechanics. Particular attention was devoted to LASr, alongside additional phasic strain components and indices of myocardial deformation.
Considering the observational design of the included studies, clinical endpoints and prognostic determinants were analyzed descriptively rather than quantitatively. Identified predictors were interpreted within their underlying pathophysiological context and categorized into key domains, including left atrial function, left ventricular performance, valvular severity, clinical characteristics, and circulating biomarkers. The frequency with which these variables were reported across studies was also evaluated to highlight the most consistently observed prognostic markers.
Where available, effect size estimates (hazard ratios or odds ratios) and study-specific LASr threshold values were reported descriptively to provide an overall perspective on the prognostic relevance of impaired left atrial function.
No formal meta-analytic pooling, heterogeneity testing, or assessment of publication bias was undertaken. Nonetheless, the consistency, direction, and reproducibility of associations across studies were carefully examined to support the validity of the overall interpretation.
All analyses were conducted at the study level. Data handling and aggregation were performed using standard spreadsheet software (Microsoft Excel, Microsoft Corporation, Redmond, WA, USA).

Subgroup analysis N/A.

Sensitivity analysis N/A.

Language restriction No.

Country(ies) involved Italy.

Keywords aortic stenosis; left atrial reservoir strain; risk stratification; prognosis; echocardiography; cardiac computed tomography.

Contributions of each author
Author 1 - Andrea Sonaglioni - Author 1 drafted the manuscript.
Email: sonaglioniandrea@gmail.com
Author 2 - Massimo Baravelli - Supervision.
Email: massimo.baravelli@multimedica.it
Author 3 - Giulio Francesco Gramaglia - The authors contributed to the search strategy.
Email: giulio.gramaglia@unimi.it
Author 4 - Gian Luigi Nicolosi - The author read, provided feedback and approved the final manuscript.
Email: gianluigi.nicolosi@gmail.com
Author 5 - Michele Lombardo - Supervision.
Email: michele.lombardo@multimedica.it

Sonaglioni et al. INPLASY protocol 202640099. doi:10.37766/inplasy2026.4.0099 Downloaded from https://inplasy.com/inplasy-2026-4-0099/
